# The weighted DMFT (W-DMFT) model: A conceptual framework for severity-adjusted caries assessment in epidemiology

**DOI:** 10.1007/s44445-026-00161-z

**Published:** 2026-05-14

**Authors:** Necmi Namal

**Affiliations:** Faculty of Medicine, Department of Public Health, Beykent University, Istanbul, Turkey

**Keywords:** Dental caries, DMFT, Weighted index, Severity assessment, Oral epidemiology, Conceptual framework

## Abstract

To critically examine the structural limitations of the classical Decayed–Missing–Filled Teeth (DMFT) index and to propose a biologically structured, severity-adjusted conceptual framework through the Weighted DMFT (W-DMFT) model. This study presents a conceptual–analytic framework without quantitative field data. The methodological constraints of the DMFT index were examined across four domains: biological progression, functional impact, radiographic detectability, and restorative assessment. Based on the enamel–dentin–pulp hierarchy, a structured weighting system (0–3) was developed to reflect increasing biological invasion and functional consequences.The W-DMFT score is expressed as a severity-adjusted mean value per tooth. The classical DMFT index applies uniform scoring that does not differentiate lesion depth or functional severity. In contrast, the W-DMFT model introduces hierarchical weighting to capture biological progression, incorporates radiographically detectable dentinal involvement, and numerically represents functional impairment. The model provides a theoretically enhanced framework for severity-sensitive caries assessment. The W-DMFT model offers a structured conceptual extension of the classical DMFT by integrating lesion depth and functional considerations into epidemiological scoring. While biologically grounded, empirical validation studies are required to assess reliability, reproducibility, and population-level performance.

## Introduction

Dental caries continues to represent one of the most widespread chronic diseases globally, exerting substantial biological, functional, and economic impact on populations. The Global Burden of Disease (GBD) Study has consistently identified untreated caries in permanent teeth among the most prevalent health conditions worldwide, underscoring its enduring public health significance (GBD 2017 Oral Disorders Collaborators 2020).

For many decades, the Decayed–Missing–Filled Teeth (DMFT) index has functioned as the principal epidemiological measure for quantifying cumulative caries experience (Klein et al. 1938). Its widespread adoption is largely attributable to its operational simplicity, reproducibility across populations, and suitability for large-scale surveillance (Broadbent and Thomson 2005). As a result, the DMFT index has become deeply embedded within international oral health reporting systems.

However, advances in caries biology and diagnostic methodology have prompted renewed examination of the structural assumptions underlying traditional indices (Pitts and Ekstrand 2013; Lewis 1996). A central methodological concern involves the uniform attribution of equal numerical weight to lesions of markedly different biological depth. Within the DMFT framework, minimal enamel involvement and advanced pulpal pathology contribute identically to the aggregate score (Broadbent and Thomson 2005; Marthaler 2004). Such numerical equivalence may obscure clinically meaningful gradients of disease severity and potentially distort interpretations of treatment need (Featherstone 2000; Bagramian et al. 2009).

In addition, although radiographic findings may be incorporated operationally, lesion depth is not intrinsically encoded within the DMFT scoring structure (Pitts and Ekstrand 2013; Ekstrand et al. 2007). This limitation may reduce sensitivity in detecting interproximal or dentin-level involvement when standardized imaging protocols are inconsistently applied. Furthermore, functional deterioration—such as extensive structural compromise, endodontic treatment indication, or loss of tooth integrity—is not differentiated within the classical scoring architecture (Marcenes and Sheiham 1993; Namal et al. 2005; Namal and Sheiham 2008). The “Missing” component similarly aggregates tooth loss without mandatory etiological clarification, potentially influencing epidemiological interpretation (Broadbent and Thomson 2005).

Contemporary caries assessment systems, including ICDAS, CAST, PUFA, and ICCMS, demonstrate that hierarchical lesion staging and clinical consequence coding can be implemented with acceptable reliability (Pitts and Ekstrand 2013; Frencken et al. 2011; Monse et al. 2010; Ismail et al. 2015; Braga et al. 2009). Nevertheless, these systems primarily operate at surface- or lesion-level and are not routinely structured as person-level aggregate epidemiological indices (Ismail et al. 2015; Braga et al. 2009).

The Weighted DMFT (W-DMFT) model is therefore proposed as a conceptual extension rather than a replacement of the classical index. By integrating enamel–dentin–pulp progression into a hierarchical weighting system, the framework seeks to preserve epidemiological compatibility while introducing severity-sensitive differentiation within a person-level aggregate metric. In doing so, the model aims to stimulate methodological discussion regarding severity-adjusted epidemiological scoring while maintaining continuity with established surveillance paradigms.

## Materials and methods

### Study design

This manuscript presents a theoretical and conceptual framework without primary clinical or epidemiological data. The objective was to construct a biologically structured modification of the classical DMFT index that integrates severity differentiation while maintaining compatibility with person-level epidemiological reporting.

### Conceptual foundation

Development of the W-DMFT framework was informed by established discussions in the literature regarding:

- Structural and analytical constraints of the DMFT index (Broadbent and Thomson 2005; Marthaler 2004).
- Hierarchical lesion classification systems (Pitts and Ekstrand 2013; Frencken et al. 2011; Monse et al. 2010; Ismail et al. 2015; Braga et al. 2009).
- Radiographic incorporation in caries detection (Pitts and Ekstrand 2013; Ekstrand et al. 2007).
- Composite indicators reflecting functional and structural oral health (Marcenes and Sheiham 1993; Namal et al. 2005; Namal and Sheiham 2008; Sheiham and Maizels 1987).

The biological sequence of caries progression—enamel involvement, dentinal extension, and pulpal compromise—served as the conceptual backbone for hierarchical differentiation.

### Core principles guiding model construction

#### Biological depth principle

Increasing tissue penetration reflects progressive biological disruption and greater therapeutic complexity. Consequently, deeper structural involvement warrants proportionally higher weighting within the aggregate score (Featherstone 2000; Ekstrand et al. 2007).

#### Functional impact principle

Advanced lesions associated with pulpal pathology, endodontic treatment need, or significant structural loss are indicative of functional compromise and long-term risk (Table 1).

**Table 1:** Hierarchical weighting structure of the W-DMFT model

Score	Biological Status	Clinical Interpretation	Functional Implication
0	Sound tooth	No detectable caries or restoration	Fully functional
1	Enamel-level lesion or minor restoration	Initial structural involvement	No major functional compromise
2	Dentinal involvement or extensive dentin-level restoration	Moderate structural disruption	Reduced structural integrity
3	Pulpal involvement, endodontic treatment, severe structural destruction, or extraction indication	Advanced irreversible damage	Functional impairment present

#### Diagnostic inclusion principle

Radiographically detectable dentinal and interproximal lesions were incorporated conceptually to enhance epidemiological sensitivity, particularly in contexts where standardized imaging protocols are applied (Pitts and Ekstrand 2013; Ekstrand et al. 2007).

### Development of the weighting structure

The hierarchical order reflects biological progression rather than arbitrary numerical assignment. The model does not claim empirical calibration at this stage; instead, it provides a biologically grounded theoretical structure pending validation.

### Calculation framework

The W-DMFT score is calculated as:

$$W-DMFT=\left(\Sigma {w}_{i}\right)/n$$

where: wᵢ represents the assigned weight for each tooth, n represents the total number of assessed teeth.

Unlike the classical DMFT, which produces a cumulative count per individual, the W-DMFT represents the average severity burden per tooth. It is therefore conceptualized as a complementary metric rather than a direct numerical substitute for traditional DMFT counts.

### Radiographic standardization and calibration considerations

When applied in empirical settings, the framework assumes standardized diagnostic protocols. Bitewing radiographs are recommended for detecting interproximal and early dentinal involvement, while periapical imaging may assist in identifying pulpal or periapical pathology.

To ensure consistency, examiner training should include calibration sessions and evaluation of intra- and inter-examiner agreement using reliability statistics such as kappa coefficients. Clear diagnostic thresholds would be required to minimize variability in severity assignment.

## Results

### Structural constraints of the classical DMFT index

- **Biological Compression:** Uniform scoring aggregates biologically heterogeneous conditions into equivalent numerical units (Broadbent and Thomson 2005; Marthaler 2004).
- **Absence of Depth Encoding:** Enamel, dentin, and pulpal involvement are not differentiated within the classical structure.
- **Limited Functional Representation:** Advanced pathology and irreversible damage are not explicitly distinguished within the scoring system.
- **Lack of Structural Radiographic Integration:** Lesion depth is not intrinsically embedded within the scoring framework, even when radiographic findings are operationally considered (Pitts and Ekstrand 2013; Ekstrand et al. 2007).
- **Etiological Ambiguity of Tooth Loss:** The “Missing” component does not mandate causal differentiation, potentially influencing epidemiological interpretation.

### Conceptual contributions of the W-DMFT model

- Hierarchical biological differentiation within aggregate person-level scoring
- Severity-adjusted epidemiological expression at the individual level
- Compatibility with standardized radiographic inclusion
- Explicit representation of functional compromise
- Alignment with treatment complexity and biological irreversibility

## Discussion

The present study does not seek to displace the historical relevance of the DMFT index; rather, it critically examines the structural logic upon which the index is constructed. The DMFT was developed within an epidemiological paradigm primarily focused on cumulative disease presence. Its longevity and widespread adoption are largely attributable to operational simplicity and reproducibility across diverse populations. However, contemporary caries science increasingly emphasizes gradation of lesion severity, biological progression, and functional consequence (Pitts and Ekstrand 2013; Pitts et al. 2017).

A central structural characteristic of the classical DMFT index is the aggregation of biologically heterogeneous conditions under a uniform scoring value. Although this facilitates comparability across populations and time periods, it may obscure clinically meaningful distinctions between reversible enamel involvement and advanced pulpal destruction. Such compression does not invalidate the index but may limit its interpretive depth when severity distribution becomes analytically relevant, particularly in settings where treatment prioritization and resource allocation depend on disease staging.

The W-DMFT framework attempts to introduce hierarchical differentiation while maintaining compatibility with person-level epidemiological aggregation. By aligning numerical weights with established biological progression—enamel to dentin to pulp—the model proposes a severity-sensitive extension rather than a competing classification system. In this sense, W-DMFT is positioned as a methodological refinement designed to complement, rather than replace, traditional surveillance metrics.

It must be emphasized that the present study remains theoretical. No clinical dataset, population-based sampling, or statistical validation has been conducted. Consequently, the proposed advantages of W-DMFT should be interpreted as conceptual propositions rather than empirically demonstrated outcomes. Future research should evaluate reliability, reproducibility, construct validity, predictive performance, and responsiveness to intervention in diverse population settings. Empirical calibration studies will be essential to determine weighting stability and inter-examiner agreement under standardized diagnostic protocols.

## Conclusion

The W-DMFT model is presented as a biologically structured, severity-adjusted conceptual extension of the classical DMFT index. By introducing hierarchical differentiation aligned with enamel, dentin, and pulpal involvement, the framework seeks to integrate lesion depth and functional consequence into person-level epidemiological aggregation.

While preserving compatibility with established surveillance paradigms, the proposed framework introduces a severity-sensitive dimension that may enhance interpretive depth in epidemiological reporting. Nevertheless, empirical validation, examiner reliability assessment, and population-based performance testing will be essential prior to clinical or surveillance implementation.

The W-DMFT is therefore positioned as a conceptual model intended to stimulate further methodological refinement and research in severity-adjusted caries assessment.

## Data Availability

No datasets were generated or analysed during the current study.
